# Well-Being and Arthritis Incidence: The Role of Inflammatory Mechanisms. Findings From the English Longitudinal Study of Ageing

**DOI:** 10.1097/PSY.0000000000000480

**Published:** 2017-06-09

**Authors:** Judith A. Okely, Alexander Weiss, Catharine R. Gale

**Affiliations:** From the Centre for Cognitive Ageing and Cognitive Epidemiology (Okely, Gale), Department of Psychology, University of Edinburgh, Edinburgh, UK; Department of Psychology (Weiss), University of Edinburgh, Edinburgh, UK; and MRC Lifecourse Epidemiology Unit (Gale), University of Southampton, Southampton, UK.

**Keywords:** arthritis, inflammation, longitudinal study, mediation, well-being, **BMI** = body mass index, **CASP** = control, autonomy, self-realization and pleasure, **CI** = confidence interval, **CRP** = C-reactive protein, **CVD** = cardiovascular disease, **ELSA** = English Longitudinal Study of Ageing, **HR** = hazard ratio, **IQR** = interquartile range

## Abstract

**Objective:**

Higher levels of well-being are associated with lower levels of inflammatory markers in healthy populations; however, it is unclear whether this association translates into a reduced risk of disease. In the current study, we tested whether the association between well-being and inflammation results in a lower risk of arthritis.

**Methods:**

The sample consisted of 5622 participants 50 years or older from the English Longitudinal Study of Ageing and included six waves of data collection. We used a structural equation modeling approach to test whether inflammatory markers (C-reactive protein [CRP] or fibrinogen) mediated the association between well-being and arthritis risk for a 10-year follow-up period.

**Results:**

Higher levels of well-being were associated with a decrease in arthritis risk (hazard ratio = 0.97 per unit, 95% confidence interval = 0.96 to 0.98, *p* < .001). Of the two inflammatory markers, only CRP was associated with arthritis risk. Mediation analysis revealed that the indirect effect of well-being (at wave 1) on arthritis risk via CRP (at wave 2) was significant (hazard ratio = 0.996, 95% confidence interval = 0.995 to 0.998, *p* < .001). This effect remained significant after adjustment for demographic and health behavior variables and depressive symptoms.

**Conclusions:**

CRP accounts for a small proportion of the association between well-being and a reduced risk of arthritis.

## Abstract 2

Supplemental digital content is available in the text.

## INTRODUCTION

After the finding that well-being is predictive of health outcomes such as disease risk and longevity (1–4), a number of studies have explored the possibility that well-being directly affects biological processes relevant to disease risk (5). Here, we focus on the link between well-being and inflammatory processes. Several cross-sectional studies have documented an association between high well-being or optimism and lower levels of inflammatory markers including interleukin 6, C-reactive protein (CRP) fibrinogen and homocysteine (6–11). These associations are not fully accounted for by differences in demographic factors, depressive symptoms, or health behaviors—suggesting that well-being may directly affect inflammatory systems, potentially, via prefrontal and limbic system pathways (5).

The link between well-being and inflammation may be clinically significant because elevated markers of inflammation in older adults are associated with a higher risk of disease and disability (12). However, the extent to which the association between well-being and inflammatory processes accounts for the relationship between well-being and disease risk remains to be explored. In the current study, we tested whether the association between high well-being and lower levels of inflammation translates into a reduced risk of one particular disease—arthritis.

We chose to examine the link between well-being and risk of arthritis for two reasons. Firstly, inflammation is implicated in the etiology and progression of rheumatoid and osteoarthritis (13–16). Thus, down regulation of inflammatory processes associated with high well-being could result in a reduced disease risk. Secondly, in a previous study, we found evidence of an association between well-being and arthritis risk (17). Using data from the Survey of Health Ageing and Retirement in Europe, we found that higher well-being was associated with a reduced risk of arthritis for a 9-year follow-up period. This association remained significant although attenuated after adjusting for demographic variables, depressive symptoms, comorbidities, and health behaviors—suggesting that additional (potentially psychobiological) mechanisms underlie the association between well-being and arthritis risk. Here, we tested this hypothesis; specifically, we examined whether the association between well-being and arthritis risk is partly mediated by the effect of well-being on biomarkers of inflammation.

The English Longitudinal Study of Ageing (ELSA) is a representative sample of men and women aged 50 years or older living in England. This data set includes measures of two inflammatory biomarkers that have previously been related to arthritis onset or progression: CRP (14,18) and fibrinogen (19). The ELSA data set also includes a measure of well-being (control, autonomy, self-realization and pleasure 19 [CASP-19]). Research into the association between well-being and health has been informed by three distinct measures of well-being: evaluative well-being (life satisfaction), hedonic well-being (feelings of joy or happiness), and eudemonic well-being (sense of purpose in life) (20). The CASP-19 is designed to assess hedonic and eudemonic well-being; higher CASP-19 scores have previously been associated with lower levels of CRP and fibrinogen in women (9), and an abridged (12-item) version of the CASP has been found to predict arthritis risk (17).

Our aim was to test whether levels of CRP or fibrinogen mediated the association between well-being and incident arthritis. The ELSA data set currently consists of six waves of data collection. We predicted that the association between well-being at wave 1 and incident arthritis (for the follow-up period) would be mediated by biomarker concentrations at wave 2. In addition, we predicted that change in well-being for the 6 waves would be associated with arthritis risk and that this association would be mediated by change in biomarker levels.

## METHODS

### Study Sample

ELSA participants are 50 years or older and were initially recruited from the Health Survey for England database in 1998, 1999, and 2001. At wave 1 (2002–2003), 11,391 core participants were recruited; since then, participants have been interviewed biennially. Refreshment samples drawn from the Health Survey for England were added at waves 3 and 4 to maintain the representation of people aged 50 to 75 years. Currently, there are six waves of data available (2002–2012). In addition to the main interview, blood samples were taken in waves 2, 4, and 6 during a separate nurse visit. Ethical approval for all ELSA waves was provided by the London Multicentre Research and Ethics Committee. All participants gave written informed consent (21).

A total of 5622 participants were included in our sample. Participants were excluded if they reported a history of arthritis or did not know whether their conditions had been diagnosed with arthritis at wave 1 (*n* = 3721) (we excluded these participants so that the well-being measure preceded arthritis diagnosis). We also excluded participants if they had missing covariate data at wave 1 (*n* = 2048). See Figure S1 (Supplemental Digital Content 1, http://links.lww.com/PSYMED/A395) for a summary of how we derived our sample. See Table S1 (Supplemental Digital Content 1, http://links.lww.com/PSYMED/A395) for a comparison of covariates among included and excluded participants. Compared with excluded participants, participants included in our sample were younger, reported fewer depressive symptoms, were wealthier, were more likely to be female, had a higher body mass index (BMI), were more physically active, drank more frequently, were more likely to have a partner, had more years of education, were less likely to report a history of diabetes, cardiovascular disease (CVD), and more likely to report a history of hypertension.

We did not exclude participants with missing CASP-19 or CRP data. Mplus uses all available data to estimate the model using full information maximum likelihood. This approach to handling missing data is recommended over listwise deletion, pairwise deletion, and similar response pattern imputation (3).

### Well-Being

Well-being was assessed at each wave with the CASP-19 quality of life questionnaire (22). The CASP-19 is designed to measure well-being across the subdomains of control, autonomy, self-realization, and pleasure. Participants respond to 19 questions on a four-point Likert scale (scored 0–3). Possible scores range from 0 to 57 with higher scores indicating higher well-being. For the study sample, the internal consistency reliability at wave 1 was high (α = 0.86).

### Inflammatory Biomarkers

Participants who were not taking anticoagulant drugs and did not have clotting or bleeding disorders were invited to provide a blood sample. Fasting samples (no food or drink except water for the past 5 hours) were taken where possible (44% of the blood samples taken at wave 2 were fasting samples). Samples were assayed for high-sensitivity CRP and fibrinogen at the Royal Victoria Infirmary, Newcastle-upon-Tyne, United Kingdom. CRP concentration was measured in milligram/liter (reference range ≤ 3 mg/L (23)) and fibrinogen was measured in grams per liter (reference range = 1.45–3.48 g/L (24)). Because of its skewed distribution, we log transformed the CRP measure.

### Incident Arthritis

At wave 1, participants were asked whether a doctor had ever told them that they had “arthritis or rheumatism.” Participants reported the month and year of their diagnosis. In subsequent waves, participants were asked to report whether they had been diagnosed with arthritis or rheumatism since their last interview. If a new diagnosis was reported, participants reported the month and year of diagnosis.

### Covariates

We chose to adjust for factors that could account for the association between well-being and arthritis risk. These covariates were the following: age, sex, depressive symptoms, socioeconomic status, level of education, relationship status, health behaviors (physical activity, alcohol consumption, and smoking status), and BMI. These factors have previously been linked with well-being (20,25–28), arthritis risk (29–33), and CRP levels (34–36). We additionally adjusted for prevalent hypertension, diabetes, and CVD at wave 1 because these conditions commonly co-occur with arthritis (37) and have been linked to lower well-being (38).

The eight-item version of Center for Epidemiological Studies Depression Scale was used to assess depressive symptoms (39). Socioeconomic status was indexed by total household wealth, which has been identified as the most accurate indicator of long-term socioeconomic circumstances in ELSA (40). Education was categorized on the basis of the highest reported level of qualification: less than O level or equivalent, O level or equivalent, A level or equivalent, higher than A level but below degree, and degree level (the US equivalent qualifications are the high school diploma for O level and 1 year of study at college or university with a B average for A level). Relationship status was dichotomized as having (coded 1) or not having (coded 0) a partner. Participants reported the frequency with which they engaged in vigorous, moderate, and mild exercise. Response options were “more than once a week,” “once a week,” “one to three times a month,” and “hardly ever or never.” As previously reported (41), responses to physical activity questions were recorded as either once a week (or more) or less than once a week. We then created the following four categories: physical inactivity, mild but not moderate/vigorous activity at least once a week, moderate but not vigorous physical activity at least once a week, and vigorous physical activity at least once a week. Frequency of alcohol consumption was recorded. Response options were the following: twice a day or more, daily or almost daily, once or twice a week, once or twice a month, special occasions only, and not at all. Participants reported their smoking status as either nonsmoker, ex-smoker, or current smoker. BMI was derived from height and weight measures taken during the nurse visit at wave 0, which took place between 1998, 1999, and 2001 (there was no BMI measure at wave 1).

To summarize, we used well-being measures from waves 1 to 6, CRP and fibrinogen measures from waves 2, 4 and 6, a BMI measure from wave 0, and all other covariate measures from wave 1.

### Analysis

We ran preliminary analysis to establish whether log-CRP or fibrinogen levels at wave 2 were associated with arthritis risk in our sample. Each biomarker was entered separately into a Cox proportional hazards model, which was additionally adjusted for age and sex. Only CRP was a significant predictor of arthritis risk (*p* = .001). Consequently, we only tested for mediation using CRP.

To examine the association between CASP-19 or CRP with arthritis risk, we ran a Cox proportional hazards model predicting arthritis risk that included age, sex, and latent variables representing well-being and CRP initial status (intercepts) at wave 1 and wave 2, respectively, and amount of change (slopes) in well-being and CRP for the follow-up period (42). We used unstandardized CRP and well-being scores in line with the recommendation by Seltzer et al, (43). Unstandardized parameter estimates are in the units of the original scale. The CASP-19 slopes were defined so that slopes represented the predicted amount of change in CASP-19 score every 2 years (between waves). Slopes ranged from −4.65 to 1.86 (M [SD] = −0.69 [0.57]). CRP slopes represented the predicted amount of change in log-CRP concentration every 4 years (between waves 2, 4, and 6). These slopes ranged from −0.47 to 0.32 (M [SD] = −0.03 [0.05]).

We ran mediation analysis testing two possible mediation pathways. Specifically, we tested whether the association between well-being at wave 1 and arthritis risk was mediated by CRP concentrations at wave 2 and whether the association between change in well-being and arthritis risk was mediated by change in levels of CRP. Mediation analyses were conducted in Mplus Version 7.4 (44) using a maximum likelihood robust estimator and Monte Carlo integration. We tested for mediation using a structural equation modeling approach (45). This allowed us to estimate the direct effect of CASP-19 (intercept or slope) on arthritis risk and the indirect or mediated effect of CASP-19 (intercept or slope) on arthritis risk through CRP (intercept or slope). Mplus uses the Delta method (46) to calculate indirect effects and provides standard errors, confidence intervals (CIs), and significance tests.

We repeated these analysis additionally adjusting for wealth, education, relationship status, depressive symptoms, health behaviors, BMI, and comorbidities.

To test for the effect of reverse causation (specifically, undiagnosed arthritis at baseline affecting well-being and diagnosis of arthritis predating first CRP measurement), we re-ran the analysis excluding participants diagnosed with arthritis at wave 2.

To test whether we would find similar results for a shorter period, we repeated the analysis using only data collected between waves 1 and 4. Because there were only two measures of CRP within this period, we could not estimate CRP slope in this analysis. Instead, we created a measure of residual change in CRP between waves 2 and 4. This was achieved by regressing CRP at wave 4 on CRP at wave 2 with adjustment for follow-up duration, the standardized residuals from this analysis were used as the residual change measure (47). We then ran the Cox proportional hazards model outlined above replacing CRP slope with the residual change measure. The sample size (*n* = 2071) for this analysis was smaller because we excluded participants with missing CRP (at waves 2 or 4).

Coefficients of log-transformed dependent variables were back transformed using the formula (*e*^β1^ − 1) × 100 and interpreted as the average percentage change in the dependent variable according to a unit increase in the independent variable. Log-transformed independent variables were back transformed using the formula β_1_ × ln(1.01) and interpreted as the amount of change in the dependent variable according to a 1% increase in the independent variable (48).

## RESULTS

There were 1090 incident cases of arthritis between waves 2 and 6. Table 1 shows the number of new diagnoses reported at each wave as well as the mean CASP-19 score (at waves 1–6) and CRP concentration (milligram per liter) (at waves 2, 4, and 6).

**TABLE 1:**
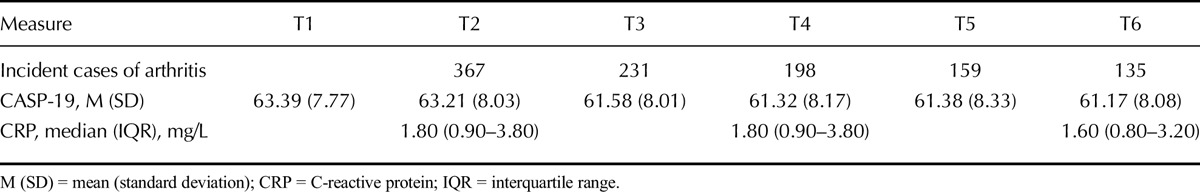
Incident Cases of Arthritis, Mean CASP-19 Score, and Median CRP Concentration at Each Wave

Table 2 shows baseline characteristics of the sample (*n* = 5266) according to well-being tertile. People with high well-being tended to be younger, wealthier, have a partner, were more likely to be female, more educated, more physically active, consumed more alcohol, and had lower depressive symptom scores. People with high well-being were also less likely to be overweight, smoke, or report a history of diabetes, hypertension, or CVD.

**TABLE 2:**
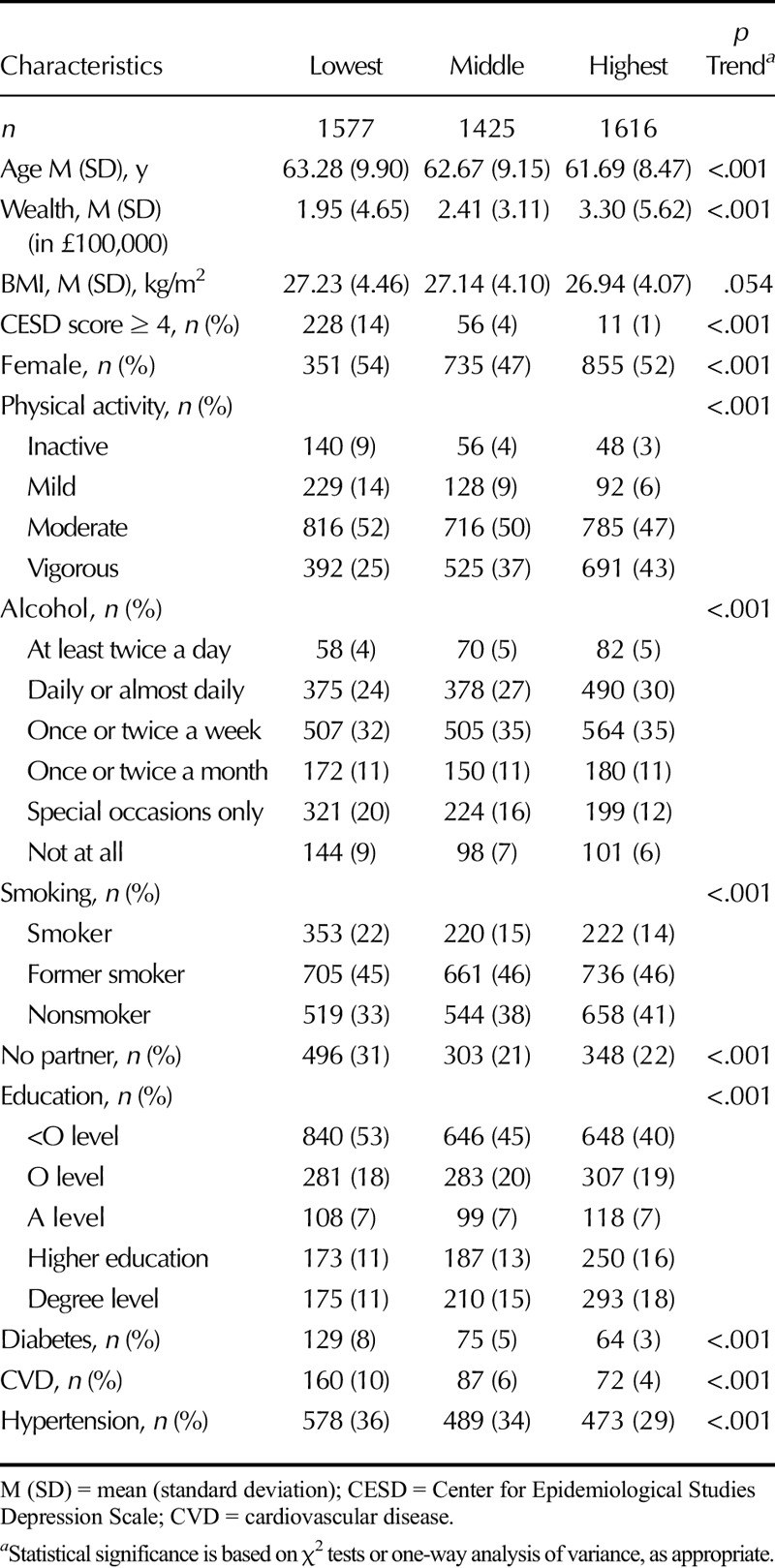
Baseline Characteristics Stratified According to Tertiles of CASP-19 Scores (Lowest, Middle, and Highest Subjective Well-Being)

In preliminary Cox models adjusted for age and sex, fibrinogen was not associated with arthritis risk (for a unit increase in fibrinogen the hazard ratio [HR] was 1.05, 95% CI = 0.94 to 1.16, *p* = .39); however, higher levels of log-CRP were significantly associated with increased risk (HR = 1.14, 95% CI = 1.08 to 1.23, *p* < .001). Difference in median CRP concentration between participants that developed arthritis (median *=* 2.00 mg/L) and those that did not (median *=* 1.70 mg/L) was significant (*p* < .001) and similar in magnitude to the differences reported by Karlson et al. (13) and Nielen (15).

**FIGURE 1:**
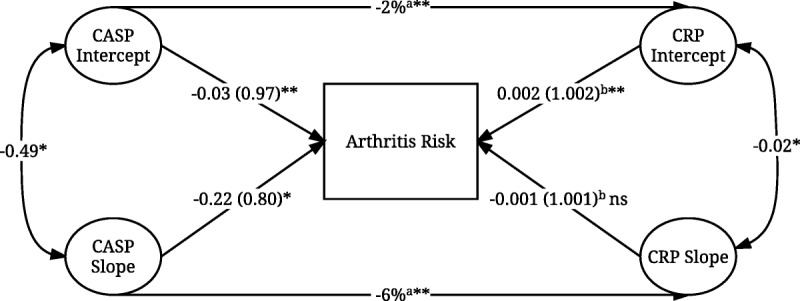
Path model adjusted for age and sex. Numbers in parentheses are exponentiated path coefficients (HRs). ^*a*^Coefficients have been transformed to represent percentage change in CRP intercept or slope according to a unit increase in CASP intercept or slope. ^*b*^Coefficients have been transformed to represent increase in arthritis risk according to a 1% increase in CRP intercept or slope. *p < .05, **p < .001, ns = *p* ≥ .05.

In the age- and sex-adjusted model (Fig. 1), the path from CASP-19 at wave 1 to CRP at wave 2 was significant. A unit increase in CASP-19 score at wave 1 was associated with an average of 2% (95% CI = 2% to 1%, *p* < .001) decrease in CRP concentration at wave 2. The path from CASP-19 slope to CRP slope was also significant with a unit increase in CASP-19 slope associated with an average of 6% (95% CI = 9% to 5%, *p* < .001) decrease in CRP slope. CASP-19 at wave 1 and CRP at wave 2 were significant predictors of arthritis risk. A 1-point increase in CASP-19 score was associated with a 3% decrease in arthritis risk (HR = 0.97, 95% CI = 0.96 to 0.98, *p* < .001). A 1% increase in CRP concentration at wave 2 was associated with an average of 0.002% (HR = 1.002, 95% CI = 1.001 to 0.002, *p* < .001) increase in arthritis risk. CASP-19 slope was also a significant predictor of arthritis risk; a unit increase in CASP-19 slope was associated with a 20% decrease in arthritis risk (HR = 0.80, 95% CI = 0.74 to 0.91, *p* < .001). CRP slope was not a significant predictor of arthritis risk. Mediation analysis revealed that the indirect effect of CASP-19 intercept on arthritis risk via CRP intercept was significant with 1 unit increase in CASP-19 associated with a 0.004% reduction in arthritis risk (*p* < .001). However, the indirect effect of CASP-19 slope via the CRP slope was not significant. The results of this model (including fit indices) are displayed in Table S2 (Supplemental Digital Content 1, http://links.lww.com/PSYMED/A395).

**FIGURE 2:**
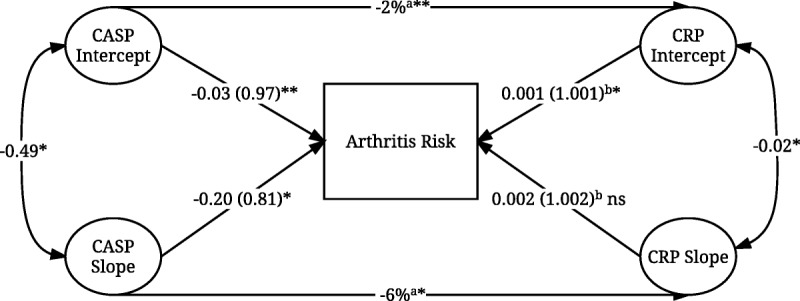
Path model additionally adjusted for comorbidities, demographic, and health behavior variables. Numbers in parentheses are exponentiated path coefficients (HRs). ^*a*^Coefficients have been transformed to represent percentage change in CRP intercept or slope according to a unit increase in CASP intercept or slope. ^*b*^Coefficients have been transformed to represent increase in arthritis risk according to a 1% increase in CRP intercept or slope. *p < .05, **p < .001, ns = *p* ≥ .05.

Estimates in the model that also adjusted for wealth, education, relationship status, depressive symptoms, health behaviors, BMI, and comorbidities were similar to those in the age and sex-adjusted model. However, the association between CRP at wave 2 and arthritis risk, which was attenuated (HR = 1.001, 95% CI = 1.000 to 1.002, *p* = .016). The association between CASP-19 slope and arthritis risk was also attenuated (HR = 0.82, 95% CI = 0.69 to 0.96). The indirect effect of CASP-19 intercept on arthritis risk via CRP intercept remained significant with a unit increase in CASP associated with a 0.002% (*p* = .020) reduction in arthritis risk (Fig. 2). The results of this model (including fit indices) are displayed in Table S3 (Supplemental Digital Content 1, http://links.lww.com/PSYMED/A395).

Additional factors significantly associated with a higher arthritis risk in the fully adjusted model, included the following: being female, being diagnosed with hypertension, having a higher depressive symptom score, and having a higher BMI.

We re-ran the age- and sex-adjusted model excluding participants diagnosed with arthritis at wave 2. Results were similar to those in the original analysis; a unit increase in CASP-19 intercept was associated with a 2% (*p* < .001) reduction in arthritis risk via the direct pathway and 0.003% reduction in arthritis risk via the indirect pathway (via CRP intercept) (*p* = .004).

In analysis using data from waves 1 to 4 only, direct pathways from CASP-19 and CRP intercepts to arthritis risk were attenuated but remained significant as did the association between CASP-19 and CRP intercept. However, the indirect pathway from CASP-19 intercept (via CRP intercept) to arthritis risk was no longer significant (*p* = .057). The indirect pathway from CASP-19 slope (via change in CRP) to arthritis risk was also not significant (*p* = .188).

## DISCUSSION

High well-being is associated with a reduced risk of developing arthritis. Our aim was to test whether CRP or fibrinogen mediated this association. Only CRP was associated with arthritis risk. Our analysis revealed that the association between well-being at wave 1 and arthritis risk for a 10-year period was partially mediated by CRP concentration at wave 2. However, it should be noted that CRP concentration accounted for only 12% of this risk association. Although change in well-being for the follow up period was associated with arthritis risk, this association was not mediated by change in CRP.

Our estimated effect size for the association between well-being and arthritis risk was similar to that found in our previous study (17), although, in contrast to this previous study, we did not detect a stronger association at younger ages.

The significant pathway between well-being at wave 1, CRP concentration at wave 2, and arthritis risk suggests that inflammatory processes are implicated in the link between well-being and arthritis risk. Although this mediation effect was modest, it supports the idea that well-being can affect disease risk via biological pathways.

Our estimate for the association between well-being and CRP is comparable with the association reported in a cross-sectional study in which an SD increase in quality of life score was associated with a 9.42% reduction in CRP concentration (6). However, as in this cross-sectional study, the direction of effect between well-being and CRP concentration is unclear. It is possible that reduction in CRP is a downstream consequence of high well-being on prefrontal and limbic system processes (49). Alternatively, it is also possible that inflammatory processes could affect well-being because they have been linked to insomnia, fatigue, hostility, and depression (50,51). It is perhaps most likely that well-being and CRP are reciprocally related; additional intervention studies could help quantify the extent to which well-being can affect CRP concentration or vice versa.

The mediation effect in our study was small due to the fact that CRP concentration at wave 2 was only weakly related to arthritis risk. Deane et al. (52) suggest that single inflammatory markers do not provide a reliable indicator of arthritis risk. This limitation may account for the weak association between CRP and arthritis risk as well as the insignificant association between fibrinogen and arthritis risk in our sample. A more accurate prediction of risk can be achieved by combining measures of multiple arthritis-related biomarkers including levels of autoantibodies and cytokines/chemokines (52). A model of the association between well-being and arthritis risk including these measures may reveal a stronger mediation effect than the one observed here.

We found that well-being and CRP slopes were significantly and inversely related—such that an increase in well-being between waves was associated with a decrease in CRP concentration between waves. The significant relationship between well-being and CRP trajectories could result from mechanisms similar to those outlined earlier. That is, change in well-being could cause a change in CRP concentration via psychobiological pathways, or change in physical symptoms (e.g., chronic pain or disability) associated with levels of inflammation could affect an individual's sense of well-being.

Change in well-being was associated with arthritis risk; however, change in CRP did not mediate this association. This is because change in CRP was not related to arthritis risk. Further work is needed to establish the timing between change in CRP concentration and the onset of arthritic symptoms (53). However, there is some indication that elevation in CRP concentration can precede the onset of symptoms by up to 20 years (14). It is possible that the 8-year follow-up period (waves 2–6) in our study was too short to capture changes in CRP concentration relevant to arthritis risk. In addition, participants who left the study before wave 6 had significantly higher levels of CRP at wave 2 than participants who remained in the study. This pattern of attrition could have resulted in an underestimation of the association between CRP or CRP change and arthritis risk in our study as participants who left may have had a higher risk of arthritis.

Analysis excluding participants diagnosed with arthritis at wave 2 yielded similar results—indicating that our findings are unlikely to reflect the effect of reverse causation, that is, undiagnosed arthritis affecting reports of well-being at wave 1. Results from analysis using a shorter follow-up period (4 years) were less consistent with our original findings. The indirect effect of well-being intercept (via CRP at wave 2) on arthritis risk was no longer significant. It is possible that this insignificant result was due to the lower power of this analysis (the number of incident cases was 421 compared with 1090 in our original analysis).

Our findings should be interpreted with caution because this study had some important limitations. Excluding a significant proportion of participants from our sample (due to missing data at wave 1) may have introduced a source of selection bias. Participants excluded from our sample differed to those included on a number of covariate variables (see Table S1, Supplemental Digital Content 1, http://links.lww.com/PSYMED/A395). In addition, arthritis incidence was ascertained using self-report. Although access to medical records would have been preferable, there is evidence that self-report of arthritis diagnosis is consistent with clinically derived measures (54). Finally, we were unable to distinguish between cases of rheumatoid arthritis and osteoarthritis. It is likely that the mechanisms underlying the association between well-being and rheumatoid or osteoarthritis are qualitatively different because these conditions involve distinct pathophysiological processes. Our study also had several strengths. The sample size was large, and we were able to control for many possible confounds.

In summary, our results indicate that CRP concentration mediates the association between well-being and arthritis risk (after taking demographic and health behavior differences into account). Although the magnitude of this mediating effect was small, we believe our findings have theoretical implications. Specifically, they provide a proof of principle that biological processes can partially mediate the link between well-being and disease. CRP concentration represents a small component of a dynamic and interactive biological system. A combination of multiple measures of biological function would enable researchers to assess the clinical significance of the pathway between well-being, psychobiological processes and disease risk (55). We hope that our findings will help motivate this line of investigation.

## Supplementary Material

SUPPLEMENTARY MATERIAL
