# Supplementary material for: Well-Being and Arthritis Incidence: The Role of Inflammatory Mechanisms. Findings From the English Longitudinal Study of Ageing
Source: Psychosom Med. 2017 Jun 9;79(7):742–8. doi: 10.1097/PSY.0000000000000480 (PMC5576535; doi:10.1097/PSY.0000000000000480)
Supplement: SUPPLEMENTARY MATERIAL [file psm-79-742-s001.pdf]

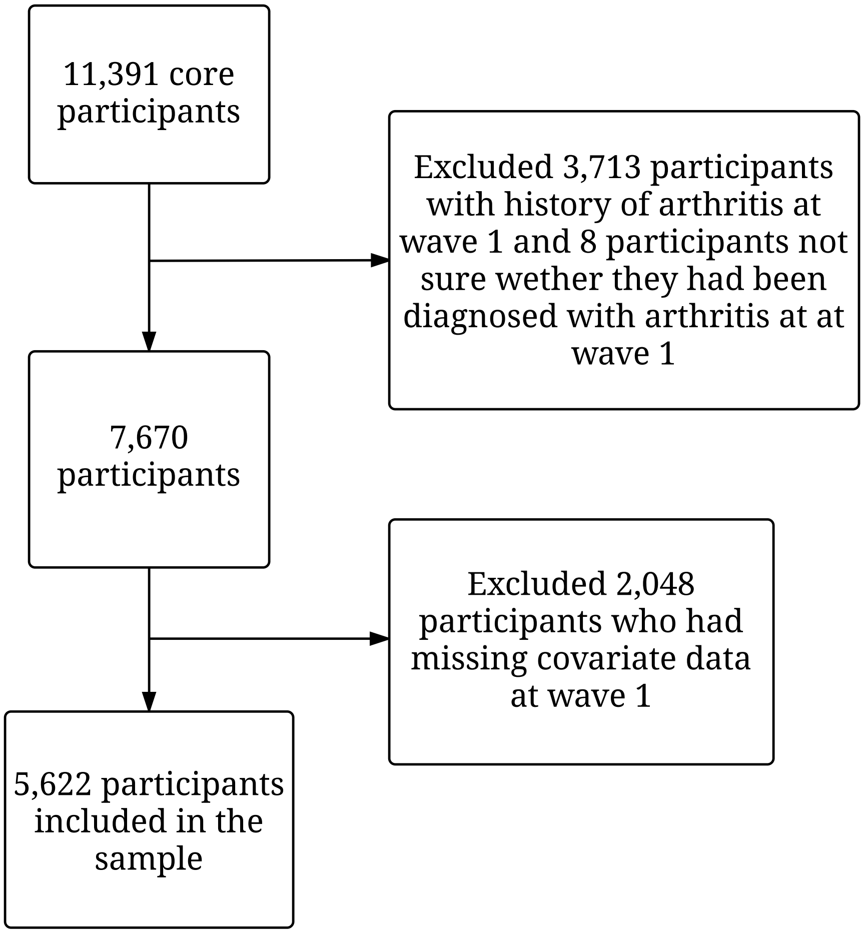

Figure S1. Flow chart showing how the analytic sample was derived.

Table S1. Baseline characteristics of participants included and excluded from analysis.

Characteristics	Included	Excluded	*p*-trend[a]
Age (yrs), *M* (SD)	63.03 (9.49)	67.19 (10.82)	<0.001
BMI (kg/m$^{2)}$, *M* (SD)	27.12 (4.25)	22.78 (12.06)	<0.001
Wealth (in £ 100,000) *M* (SD)	2.4 (4.4)	1.8 (3.2)	<0.001
CESD score *Mdn* (IQR)	617 (11)	1312 (20)	<0.001
Female, No. (%)	2613 (49)	3592 (59)	<0.001
Physical Activity, No. (%)			<0.001
Physically inactive	310 (6)	901 (15)	
Mild physical activity	558 (11)	1111 (18)	
Moderate physical activity	2634 (50)	2651 (43)	
Vigorous physical activity	1764 (33)	1288 (21)	
Alcohol consumption, No. (%)			<0.001
At least twice a day	229 (4)	260 (4)	
Daily or almost daily	1379 (26)	1281 (21)	
Once or twice a week	1768 (34)	1610 (26)	
Once or twice a month	570 (11)	587 (10)	
Special occasions only	905 (17)	1289 (21)	
Not at all	415 (8)	923 (15)	
Smoking status, No. (%)			0.022
Smoker	952 (18)	1046 (17)	
Former smoker	2365 (45)	2867 (47)	
Non smoker	1949 (37)	2040 (33)	
No partner, No. (%)	2162 (35)	1399 (27)	

Education, No. (%)			<0.001
Less than O-level or equivalent	2668 (47)	3841 (63)	
O-level or equivalent	1049 (19)	845 (14)	
A-level or equivalent	412 (7)	312 (5)	
Higher education below degree	721 (13)	577 (9)	
Degree level or equivalent	825 (15)	520 (9)	
History of diabetes, No. (%)	325 (6)	528 (8)	<0.001
History of CVD No. (%)	377 (7)	714 (11)	<0.001
History of hypertension, No. (%)	1857 (33)	2573 (40)	<0.001

[a] Statistical significance is based on χ2 tests or t-tests, as appropriate.

Table S2. Model 1: Estimates and Model Fit.

Path	Estimate	SE	*p*-value
CASP-19 intercept → CRP intercept	-0.021	0.003	<0.001
CASP-19 intercept → CASP-19 slope	0.486	0.198	0.014
CASP-19 intercept → ST	-0.030	0.005	<0.001
CASP-19 slope → CRP slope	-0.066	0.009	<0.001
CASP-19 slope → ST	-0.217	0.083	0.009
CRP intercept → CRP slope	0.023	0.010	0.025
CRP intercept → ST	0.189	0.047	<0.001
CRP slope → ST	-0.019	0.877	0.98
Age → ST	-0.002	0.003	0.56
Sex → ST	0.467	0.062	<0.001

ST = survival time, number of free parameters = 27 Akaike (AIC) = 179477.447

Table S3. Model 2: Estimates and Model Fit.

Path	Estimate	SE	*p*-value
CASP-19 intercept → CRP intercept	-0.021	0.003	<0.001
CASP-19 intercept → CASP-19 slope	-0.488	0.198	0.014
CASP-19 intercept → ST	-0.025	0.006	<0.001
CASP-19 slope → CRP slope	-0.067	0.009	<0.001
CASP-19 slope → ST	-0.203	0.085	0.017
CRP intercept → CRP slope	-0.024	0.010	0.020
CRP intercept → ST	0.121	0.050	0.014
CRP slope → ST	0.180	0.902	0.84
Age → ST	-0.001	0.004	0.75
Sex → ST	0.458	0.067	<0.001
Alcohol Consumption → ST	-0.024	0.024	0.33
Education → ST	0.007	0.023	0.76
Relationship Status → ST	-0.054	0.072	0.46
Smoking Status → ST	-0.033	0.044	0.45
Physical Activity → ST	0.032	0.040	0.42
Depressive Symptoms → ST	0.041	0.018	0.022
Hypertension → ST	0.137	0.066	0.039
Diabetes → ST	-0.045	0.131	0.73
CVD → ST	-0.010	0.125	0.94
BMI → ST	0.039	0.007	<0.001
SES → ST	-0.014	0.026	0.58

ST = survival time, number of free parameters = 38, Akaike (AIC) = 179451.044
